# Supplementary material for: The Invisibility of Scotomas I: The Carving Hypothesis
Source: Optom Vis Sci. 2023 Jul 27;100(8):515–29. doi: 10.1097/OPX.0000000000002048 (PMC10510785; doi:10.1097/OPX.0000000000002048)
Supplement: Supplementary file 1 [file ovs-100-515-s001.docx]

**ORIGINAL INVESTIGATION**

**The Invisibility of Scotomas I: The Carving Hypothesis**

Eli Peli, MS, OD, FAAO, Robert Goldstein, PhD, and Jae-Hyun Jung, PhD, FAAO

Schepens Eye Research Institute of Massachusetts Eye and Ear, Department of Ophthalmology, Harvard Medical School, Boston, Massachusetts (all authors)

Sshort title: The Invisibility of Scotomas I

no tables; 6 figures; 2 appendices; 20 SDC videos

Submitted: February 17, 2023; accepted June 6, 2023.

Corresponding author:

Eli Peli

eli_peli@meei.harvard.edu

**ABSTRACT**

**Significance:** Veridical depictions of scene appearance with scotomas allow better understanding of the impact of field loss and may improve the development and implementation of rehabilitation. Explanation and depiction of the invisibility of scotoma may lead to patients’ understanding and thus better compliance with related treatments. **Purpose:** Simulations of perception with scotomas guide training, patient education, and rehabilitation research. Most simulations incorrectly depict scotomas as black patches, though the scotomas and the missing contents are usually invisible to patients. We present a novel approach to capture the reported appearance of scenes with scotomas **Methods:** We applied a content-aware image resizing algorithm to carve out the content elided under the scotoma. With video sequences we show how and why eye movements fail to increase the visibility of the carved scotomas. **Results:** Numerous effects, reported by patients, emerge naturally from the scotoma carving. Carving-eliminated scotomas over natural images are barely visible, despite causing substantial distortions. Low resolution and contrast sensitivity at farther eccentricities and saccadic blur reduce the visibility of the distortions. In a walking scenario, static objects moving smoothly to the periphery disappear into, and then reemerge out of peripheral scotomas invisibly. **Conclusions:** Scotoma carving provides a viable hypothetical simulation of vision with scotomas, due to loss of neurons at the retinal ganglion cell level and higher. As a hypothesis, it generates predictions that lend themselves to future clinical testing. The different effects of scotomas due to loss of photoreceptors are left for follow-up work.

Understanding visual field loss is essential for the development of vision rehabilitation or restoration devices and techniques.^1^ Portions of the visual field may become insensitive to visual stimuli in various diseases at any point along the visual pathway from the retina to the cortex. Such field losses are measured clinically using perimeters, and the part of the visual field that is “blinded” is called a scotoma. The perimetric results are essential for detection and the diagnosis of diseases. If the damage is post-chiasmatic, the scotomas are typically bilateral, and they result in binocular homonymous scotomas delineating parts of the field of view that are rendered invisible with both eyes open. In pre-chiasmic diseases or injuries (optic neuropathy or retinopathy), the field losses are monocular. Monocular scotomas in both eyes may overlap in part, and the binocularly overlapping scotomatous areas result in binocular scotomas.

In this paper, we address only the perception with localized discrete scotomas surrounded by a seeing field. We do not address the perception at the edge of the normal field (i.e., the loss of the temporal crescent with loss of one eye^2^) or scotomas that extend into the limit of the normal field such as hemianopias, quadranopias, and tunnel vision. We briefly address the case of a bitemporal hemianopia that, in combination with esotropia, results in a binocular vertically elongated central scotoma mostly surrounded by seeing field.^3^

Simulations/illustrations of the perception of patients with scotomas guide clinicians’ training and patients' and caretakers' education, as well as research efforts. In almost all simulations, the scotomas are incorrectly depicted as black (or grey) patches visibly occupying part of the field and thus occluding part of the scene. In these illustrations, contents elided by scotomas are not visible, but the location, shape, size and boundaries of the scotoma are visible. In fact, the scotomas are usually not apparent and thus are “invisible” (no field occupied with black or grey space), with the contents in the scotomatous field are missing from the patient’s consciousness.^1^ The focus of this paper is this “invisibility” of the existence of scotoma. Several studies reported spatial distortions as a form of visibility of scotomas or as a consequence of the scotomas (Schuchard RA. IOVS 1991;32:ARVO abstract 816).^4-8^ Such distortions were illustrated in one simulation of central scotoma^9^ and are addressed here.

A few studies have directly tested the validity of the black patch simulations in patients with field losses and found them to be incorrect.^3,10,11^ In one study, black patch simulations of vision with early (arcuate) and late (tunnel vision) glaucomatous field loss were rejected by all 50 patients and 26% reported no visible loss.^10^ 76% of 29 patients with age-related macular degeneration (AMD) rejected the black patch simulation taken from the National Eye Institute website.^11^

Fletcher et al.^12^ found that most patients with AMD and binocular scotomas were completely unaware of a visual field loss, even with very large scotomas. Yet, 44% reported objects disappearing on them. Another paper questioned patients with severe pericentral glaucoma (within the 10-2 HFA field) about their perception of an Amsler grid.^13^ Only 5% of patients’ eye was reported seeing black patches. Many of the patients could detect missing lines in the grid or report seeing such lines as blurred or grey. Note, however, that the Amsler grid is a perimetric test and does not represent vision in a natural view of the environment.

Despite all the counterevidence, most simulations of vision with scotomas, central or peripheral, continue to depict black (or grey) patch(es) occluding parts of the scene in the patient’s view. Such simulations are presented on most websites^14-16^ and textbooks.^17-19^ The high prevalence of such wrong simulations confuses patients who may easily see that they are wrong (at least for them) and when they report that discrepancy to their relatives the confusion spreads to the caretakers.

One way to understand how scotomas can exist, be measurable by perimetry, and yet not be perceived (be “invisible”) is to consider optical scotomas caused by optical devices. An optical scotoma caused by spectacles-mounted magnifying devices, such as high-power positive spectacles lenses used to correct hyperopia, and, in particular, to correct hyperopia due to aphakia.^20,21^

These lenses were typically produced in a lenticular shape with a round central high-power lens surrounded by a plano or lower power lens used to mount the lens in the frame (Fig. 1). The lens magnifies the view through the lens, which results in a ring-shaped loss (ring scotoma) of a section of the field of view surrounding the lenticular lens that can be measured with a perimeter (Fig. 1A). Observing or photographing a scene^22^ through such a magnifying lens does not result in a black or any other colored ring, though careful examination reveals the loss of content that falls within the ring-shape scotoma surrounding the content seen through the central lenticular lens due to the magnification (Fig. 1B). The loss of content (elided content) is not difficult to note upon careful foveal examination of the photograph. In actual use, however, the elided content is most of the time located at far peripheral eccentricities (>40°). Therefore, the contents are affected by the reduced resolution and contrast sensitivity in the periphery, and by being outside the patient's attention, making the loss of content very difficult to note. Yet, under eye^20^ or head^21^ movement, objects falling into the ring scotoma (the elided details) may occasionally pop into the visible central part of the field of view and become apparent, startling the patient in what is called the “Jack-in-the-Box” phenomenon. This (optical) effect illustrates the relevance of image dynamics to the perception of the effects of scotomas, which we address further below.

A second common optical scotoma is the scotoma manifested at the apex of any prism included within the field of view, called the apical scotoma.^23^ Here too, despite the clear record of a scotoma obtained in perimetry (Fig. 1C), the view through the lens (even monocularly, with or without hemianopia) does not include an explicit black patch that occludes contents and/or occupies the field of view (Fig. 1D).^22^ Instead, the shifted image in the prisms replaces part of the unshifted image at the same location of the prisms, and the realization of content loss is cognitively implied and requires attention. The peripheral prism, which shifts a portion of the field of view for patients with homonymous hemianopia, is usually placed at more than 20° eccentricity above and below the line of sight^24^ (Fig. 1C) so that all the elided content details in the apical scotoma and visible details in the surrounding areas are subject to the reduced resolution and contrast sensitivity peripherally, making the loss of content less apparent. This eccentricity-dependent loss of resolution is not illustrated in these photos captured by a camera (Figs. 1B, 1D) but is simulated later in this paper.

In both optical scotoma examples above, the scene under the scotoma in the field of view, the elided contents, are replaced by visual content from another part of the scene through the optical devices, the magnified central area in the case of the ring scotoma, and the shifted scene in the prism case, respectively. Importantly, the scotoma, which can be appreciated cognitively, is not explicitly visible as a dark patch in the field of view, but the part of optical device that occupies the scotomatous field now shows another part of field of view.

A different type of scotoma that may be measured perimetrically yet be invisible can occur in patients with complete bitemporal hemianopia. Damage to the optic chiasm, due to disease, surgery, or trauma, results in heteronymous (bitemporal) scotomas. The residual fields in both eyes may be non-overlapping and thus may leave the binocular field of view largely intact, except for the loss of the temporal crescents bilaterally. A binocular vertical central scotoma can be measurable in patients with bitemporal hemianopia and manifest esotropia (Fig. 2A).^3,26^ Patient’s perception is inconsistent with the illustration in Fig. 2B, rather these patients report the effects shown in Figs. 2C and 2D. Note that the elided content here is not replaced by anything, it is simply eliminated (carved out). These patients’ perception can be simulated optically by changing the magnitude of the strabismus, e.g., by bifold mirrors in Fig. 2D or variable prism in the phoropter, we can vary the horizontal extent of the scotoma (i.e., the width of the elided content), resulting in perception of shrinking or widening of the contents (e.g., the face of the examiner, as in Fig. 2D). Because the vertical meridians on both sides of the scotoma represent the same visual direction (straight ahead), they are perceived to be abutting, rendering the scotoma invisible. This is a special situation, but we are hypothesizing that similar elided contents and thus carved out perceptions occur with other central and peripheral discrete scotomas surrounded by seeing retina.

The physiological blind spot is a monocular discrete scotoma that marks the optic nerve head. It is an ellipse measuring about 5°×7° centered about 15° temporally in both eyes' visual fields. Because everyone can experience the blind spot themselves, its properties and invisibility have been studied more than any other type of field loss. When covering one eye, the blind spot in the other eye becomes a binocular scotoma, yet it is not visible, though it can be demonstrated and easily measured perimetrically. It is important to consider the lessons from observations with the physiological scotoma, yet it is also important to note that it is a special scotoma, as the brain has not had a representation of the space under that scotoma except through the other eye.

While it was clear to some for centuries that scotomas are not seen by patients as black patches in the field of view, it was not obvious how they do look, let alone how to illustrate, or simulate the vision with them. Understanding and illustrating the invisibility of scotomas or the possible impact of them on the view by the remaining functional field right next to the scotoma is not simple.

Many types of scotomas are not explicitly observed and are out of awareness even when they are located centrally within the visual field. Therefore, we advance here the hypothesis that the visual pattern under the scotoma, the elided content, is *carved out* of the perceived scene and is not replaced by anything, as shown above for the special case of bitemporal hemianopia with esotropia (Fig. 2). This *carving out* requires the surrounding scene content from just outside the scotoma to somehow shift into the void created in the scene by the scotoma. In the general case, such a shift must result in local spatial distortion of the content outside the scotoma. Local distortions will interact with the image motions because of eye movements or self-motion. These interactions, which may be perceived as local motion, are addressed below.

**Scotoma Carving**

We illustrate/simulate the invisible scotomas based on the carving out of elided contents by modifying the Seam Carving image processing technique,^27^ designed as a content-aware image rescaling/resizing process. It is now widely used in image processing packages such as Photoshop (Adobe Inc., San Jose, CA). We therefore call this simulated visual perception process Scotoma Carving. The seam carving algorithm may be applied to remove a predesignated part of the image (i.e., the scotoma) by forcing these portions to be removed. This removal of contents from the designated part of the image without replacing any is the main effect resulting in the invisibility of the scotomas. It is used to illustrate the effects that most content-aware carving may cause and enable us to examine the consistency of such effects with scotomas as reported by patients and observed in studies. The use of this specific content-aware removal algorithm is not meant to directly represent the visual mechanism underlying the perception with scotoma but rather to illustrate the appearance, to a patient, of a scene with elided contents carved out. Of course, some mechanism that will provide similar effects is necessary for this hypothesis even to be considered. Preliminary validity of this simulation is supported by the consistency of the results with effects reported by patients with scotomas, as shown in the rest of this paper.

In the Seam Carving algorithm,^27^ “seams” (one-pixel wide meandering paths of least energies through the image) that progress either top to bottom (vertical) (Fig 3B) or left to right (horizontal) are calculated and later removed. A seam contains only one pixel in each row or column for the vertical or horizontal seams, respectively. The algorithm selects seams to minimize the removal of salient contents (hence content-aware). A seam is constructed by using an energy function (local contrast measure) that measures the visual saliency of the pixels. At each step, the next pixel (from among neighboring pixels in the next row for a vertical seam), the one with the lowest energy is selected. In the images produced here, the (3 × 3) Prewitt operator^28^ was used as the energy function. A seam's total energy is calculated by summing the energy calculated for each pixel. Once all the seams are constructed, seams with the lowest cumulative energy rank are removed progressively from the image one by one resulting in a reduction in the size of the image by one pixel in the orthogonal direction (i.e., horizontal size reduction of one pixel for a vertical seam removal).

The seam carving approach may be applied to preferentially remove the part of the scene underlying a scotoma. That portion of the image is pre-set to have minimal energy (or even negative energy), so seams will always pass through the scotoma and thus are removed first. The algorithm is forced to select the seams near the scotoma to pass within the scotoma while the seams portions outside of the scotoma (before and after passing through the scotoma) are free to search for the best path to remove additional (low energy/less salient) pixels to complete the seam. The lowest cumulated energy seams are removed in order until all the pixels under the scotoma are removed. More details of the scotoma carving computations applied here are in Appendix 1, available at http://links.lww.com/OPX/A621.

The scotoma carving process produces seams widely distributed across the image to prevent visible distortions but results in more distortion near the scotoma since all seams converge into it. If scotoma carving is applied using vertical seams, the right and left sides of the scotoma will be forced to close into apposition. Similarly, for horizontal seams, the regions above and below the scotoma will close. When both vertical and horizontal seams are applied, the distortions are in all directions around the scotoma. These distortions get smaller (more distributed) farther away from the scotoma. The image processed by scotoma carving ends up smaller than the original image. That reduction in the image size is by the size of the scotoma. However, if such an effect happens in natural vision, it will not be noticeable, as the visual field of view (200° horizontally) is much larger than typical scotomas (about 10°) and there is no clear visible boundary of the full visual field.

Distortions similar to those occurring with the scotoma carving algorithm have been reported by patients. Patients with small right paracentral homonymous hemianopias (Fig. 3A) reported seeing the left shoulder of a person facing them appear thinner than the right shoulder.^6^ A simulation of this paracentral scotoma using the scotoma carving illustrates a similar effect (Fig. 3). In this case, the carving was applied using vertical seams resulting in the shrinking of the horizontal shoulder line. The same patients were presented also with two vertical lines laterally shifted from a fixation target to the right and left.^6^ The right line passing through the scotoma was reported by both patients to be shorter than the left line, representing vertical distortion (shortening). We have replicated this effect by applying scotoma carving (not shown). Andrews^29^ reported that a horizontal line extending over time through the physiological blind spot appears shorter than a similar line extending at the same time outside the physiological blind spot. In this dynamic case, the difference in size was more pronounced than with the static images used by Safran^6^ and others, although the difference may have more to do with the difference in eccentricity than with dynamic. All these reported effects are consistent with the results of scotoma carving process. Other, related, spatial distortion effects were reported in patients with quadranopias due to cortical lesions (Ramachandran VS, IOVS 1992;33: ARVO abstract 1348).^8^

The selection of the direction of the seam carving to perform may be an important decision in the carving algorithm. Preferably, the direction of applied carving should follow what *may* be taking place in the visual system, but evidence for that is limited. The few examples listed above suggest that carving takes place in the direction orthogonal to the test stimulus (e.g., vertical seam carving for the width of the shoulder in Fig. 3), but in almost all clinical studies only one direction was evaluated. Because of the curved and diverging paths taken by seams outside the scotoma, the distortions, while mainly orthogonal to the direction of carving, may be affecting other directions.

Another consideration may be that the carving should take place in the direction orthogonal to the line connecting the fovea with the discrete peripheral scotoma (i.e., vertical seam carving for left and right peripheral scotomas and horizontal seam carving for upper and lower peripheral scotomas, respectively). The benefit of such a choice is that the distortions imparted by the seam path outside of the scotoma would be directed mostly away from the fovea, keeping the distortion farther in the periphery. Note that fixation is naturally aimed at higher saliency (high contrast portions of the scene) and as a result, the seams would be likely to be diverted away from the object of interest being fixated. The algorithm would result in lower distortions if the direction of the seam carving is aligned with the longer dimension of the scotoma, which would require fewer seams to be carved out. This last rule will coincide with the second rule when carving arcuate or ring scotomas, which may be the case in glaucoma and retinitis pigmentosa.

In this paper, we implemented horizontal and vertical carving and selected the seam carving directions to follow the directional selection rules above. For paracentral scotomas, we implemented such orthogonal seam carving (i.e., applying vertical seam carving for lateral scotomas and horizontal seam carving for vertical scotomas, respectively). In the case of multiple scotomas, the orthogonal carving algorithm was applied separately to each scotoma to follow that rule. For central scotoma, this last rule may not be applicable and thus we have applied alternating horizontal and vertical seam carvings. This approach is also more efficient in cases of roughly round scotomas, as is frequently the case with central scotomas. Because of the curved path of the seams, and due to the combined effect of nominally horizontal and vertical seams, the distortions outside the carved scotoma may be affected in all directions not just in the horizontal and vertical directions (as the seams may deviate by up to 45° from the primary direction).

The scotoma carving images shown in Figs. 3 and 4 result in substantial local spatial distortions of the scene outside of the carved scotomas. These distortions are noticeable when the scotoma carved image is compared side by side with the original image, as seen in these figures. Yet, without comparison with the uncarved image (not accessible by patients), these substantial distortions are barely noticeable. In addition, extended foveation of the carved image as one investigates details at different locations from the figures is not available for real patients since the scotomas are moving with changes in fixations, remaining eccentrically. Comparison of details is sometimes possible within one image, such as the difference between the two shoulders (Fig. 3).

While Fig. 3 simulates the view with a paracentral scotoma near the fovea, the images in Fig. 4 simulate the effect of farther peripheral scotomas (arcuate scotomas) located at about 15° of eccentricity and, therefore, unlikely to be noticed if foveation is maintained at the indicated fixation locations. If the images are viewed at the fixation marked in Fig. 4A under the correct display size from the appropriate viewing distance, the distortions fall at the appropriate far eccentricity, where they are very difficult to note.^30,31^ A simulation of eccentric scotomas effects can be generated by incorporating the decline in resolution and contrast sensitivity with eccentricity.^32^ Although the eccentric scotoma effects can be examined foveally over the entire image , the patients with the scotoma cannot examine them since the scotomas are moving with the foveation. This aspect is addressed further below (Fig. 5)

**The Interaction of the Scotomas with Vision Dynamics**

Objects in the scene that moves slowly into a scotoma merely disappear, yet patients do not experience the scotoma itself, which remains invisible. One can experience this by closing one eye and moving a finger through the physiological blind spot in the other eye. Here too, part of the finger disappears – but it just disappears into nothingness. Neither the scotoma nor the outline of the scotoma emerges or becomes visible. This is an example of the role of motion in perception with scotomas. There are various types of motion to be considered. The most common is the whole retinal image abrupt motion due to saccades that occurs about 3 times per second. Another commonly encountered movement is the radial optic flow of the world image across the retina in locomotion. This smooth optic flow is much faster at farther retinal eccentricities than near the focus of expansion. Another motion is the smooth sweeping of the whole static world image across the retina with the pursuit of a smoothly moving object of interest. In all three cases, the perception of a stable world is maintained despite the movements of the world image on the retina. Another motion of interest is the movement of one or a few objects of interest across the field of view while fixation is maintained on another static object of interest. This results in a mostly static retinal image except for the few moving objects. The interaction of the invisible scotoma with retinal image motions is of interest. After showing scotoma carving effects on static images, we then deal with the effects of these most common saccadic movements and optic flow on the invisibility of scotomas.

**Effects of Saccades**

Most simulations of vision with scotomas are limited to static images. The videos that we found online typically present smooth panning movements of the camera with the black (or gray) patch scotomas fixed in the frame.^33,34^ The latter reference does include a few simulations of saccadic-like movements.

Smooth pursuit eye movements require tracking a moving object, which is not the situation shown in most of these videos, except for our simulation of vision with central scotoma^35^ that included some smooth movement of the scotoma within the scene and the video frame.^36^ In a Public Broadcast Service television program, featuring a low vision device based on an early head-mounted display,^9^ the simulations also showed smooth movement of the scotoma either at the center of the frame or within the frame; however, the eye movements measured and shown in the same program were all saccades, yet these were not simulated. Tracking a smoothly moving object using peripheral vision is not easy.^37, 38^ Simulations of vision with scotomas should account also for the change in the location of scotomas across the scene with eye movement. As patients' saccade across a scene, scotomas and their associated distortions move abruptly around the scene with every saccade. The temporal changes in local distortions across a saccade result in apparent local motions that may attract attention to the distortion. However, patients do not report noticing such effects or perception of motions due to changes in local distortion during saccadic eye movements, indicating another type of invisibility of the effects of scotomas.

The simplest way to represent the effect of saccades is to switch quickly between the images in Fig. 4B and 4C as done in Video 1 (available at http://links.lww.com/OPX/A622), representing an abrupt change in fixation. When watching this video without a fixation marker, the viewer is free to look around the scene examining the difference between two frames may notice the apparent motions during switching between two images and detect the local spatial distortions.

However, if a blank or blurred frame representing saccadic suppression, or merely saccadic blur,^39,40^ is inserted between the pre- and post-saccades frames, the distortions and the apparent motions are largely masked (Video 2, available at http://links.lww.com/OPX/A623). If the observer performs a saccadic eye movement exactly when the images are switched (guided gaze-contingent), these obviously apparent image motions become invisible, because they are masked by the observer’s saccade - even without the simulated blur (not shown). We approximate gaze-contingent display using a “guided-gaze” stimulation, where the image is switched 200 ms after (i.e., an average saccadic delay) the fixation target shifted (Video 2, available at http://links.lww.com/OPX/A623).

Another way of incorporating the effects of saccades in the scotoma carving simulations is the “flipbook” presentation (see Video 3, available at http://links.lww.com/OPX/A624). Here an image larger than the angular width of the display, a panorama image (Fig. 5A) is used (representing a 109° wide scene). A series of fixation locations are selected within the larger image at various locations representing fixations of a person (with early glaucoma field loss) scanning the view with saccades. Scotoma carving is applied using the simulated patient’s scotoma positioned relative to each selected foveal fixation location. In these simulations, the same arcuate scotomas used in Fig. 4A are used but flipped upside down to represent lower arcuate scotomas (Fig. 5A). The processed image is then cropped to fit the display size (45° wide) with the fixation always in the center (Fig. 5B). The image in Fig. 5B is obtained by taking the 4^th^ fixation cross (green) from the left side of Fig. 5A. If the images are viewed on a 24” HD monitor (~20” wide) from a distance of approximately 24”, they span about 45° of visual angle. The series of these images representing a sequence of 2 sec fixations across the larger image is presented in Video 3 (available at http://links.lww.com/OPX/A624), which simulates the effect of saccadic eye movement with the central guided fixation. Because most naturally occurring saccades are smaller than 15°,^41^ there is substantial overlap between consecutively presented frames making the image shift obvious. However, if the observer maintains fixation at the center of the display, the motion artifact caused by the changing distortions between the images (which are mainly taking place at about 15° retinal eccentricities) are not visible. During this simulated fixation movement, the whole retinal image is updated, and, therefore, any changes in the images from before to after fixation are not easy to track. As a result, in this flipbook simulation video, no saccadic blurred frame was required to mask the transition. The reader may pause the video on any frame to examine the frame carefully. If the reader fixates on the fixation target, the peripheral scotoma carving is hardly noticeable due to lower resolution and contrast sensitivity of the peripheral vision. In a few frames, only the effect of one of the lateral scotomas may be noted this way and, in many frames, no scotoma is noted. The effect of the lower smaller arcuate scotoma is observable but only if one is searching for it deliberately. The motion associated with the local distortions due to this lower scotoma is never observable. We provide examples of similar processing with additional 10 images of various characteristics demonstrating that the invisibility of the scotomas the distortions and the motion artifacts are a general effect and not specific to the image in Fig. 5 (Appendix 1: Arcuate Scotoma Carving, available at http://links.lww.com/OPX/A621).

**Effect of Eccentricity**

It is well established that the contrast sensitivity declines with increased retinal eccentricity.^32^ That effect, which is easy to measure is, nevertheless, invisible to people with normal sight without steady fixation and when attempting to distinguish details in the periphery. Under casual observation, people perceive their full field of view to be sharp in focus and at high contrast. Peli et al.^32^ applied the pyramidal multi-scale vision model^42^ to simulate the effect of reduced contrast sensitivity with retinal eccentricity. The validity of these simulations was then verified using wide angle natural images.^43^ This effect combined with scotoma carving was applied here in Fig. 5C, Figs. 6C&D, and Fig. 7. In inspecting those scotoma carved images either with fixation maintained at the center of the image or with the simulation of the effect of eccentricity (Fig. 5C), it is difficult to note the image details carved out without prior examination and comparison with the outlined scotoma image. Removal of the image regions within the scotomas using the carving algorithm results in spatial distortions around the elided areas (for example, straight lines becoming curved). Distorted spatial details are visible when affecting familiar objects like faces, but even in these cases, the distortions are frequently difficult to discern without comparing the processed to the original image.

**The Effect of Optic Flow**

When a patient with peripheral (arcuate) scotoma(s) is walking or driving, the image of the scene sweeps smoothly across the retinas spreading peripherally from the focus of expansion, which is at the direction of the movement. Static objects near the focus of expansion move/expand very slowly. Static objects at farther eccentricity from the focus of expansion move faster than the objects near the focus of expansion as they move farther peripherally. A patient walking straight may be looking in any direction, but assuming, first, fixation at the focus of expansion (note the first and third fixation markers in Video 4, available at http://links.lww.com/OPX/A625), static objects at the periphery will move away to the farther periphery following the optic flow and thus smoothly move into and then out of the peripheral scotomas (Video 4, available at http://links.lww.com/OPX/A625). When a peripheral object (or parts of an object) enters and later reemerges from the scotomas with no eye movement, the change in the object's appearance (similar to “Jack-in-the-box” phenomenon) may be expected to be visible, as it is not masked by saccadic blur. Yet, patients with such peripheral scotomas do not report such changes, just as they do not report changes in static images with or without saccadic eye movements. Normally sighted readers may experience the effect by walking while looking straight ahead with one eye closed. The optic nerve head scotoma in the other eye is at about the same eccentricity (15°) and of similar size (5°x7°) as the early arcuate scotomas in glaucoma yet the physiological scotoma remains invisible during this movement. This invisibility of the scotomas may be accounted for by the lack of attention to the periphery and by the lower resolution at that peripheral eccentricity. It is also the case that the visual system is accustomed to such disappearance and reemergence of objects during walking, as a closer object may temporarily occlude a farther object, which will then reappear farther peripherally. Thus, the effect of the lateral scotomas is similar to the familiar occlusion imparted by the closer objects within the optic flow during walking. Note that the objects so occluded, or covered by the scotomas, may be static or may be moving on their own within the scene.

The walking patient is likely looking at the focus of expansion, but nothing prevents the walking patient from looking elsewhere from time to time. In most cases, the shifts of gaze will be saccadic. In these cases, the effect of saccadic blur will be similar to the effect discussed above and thus further contributes to the invisibility of the scotoma or the local motion due to changes in peri-scotomas distortions.

As the gaze is shifted laterally away from the focus of expansion and lands on a peripheral static object in the environment, that object will appear to move laterally triggering smooth pursuit eye movements to track the object with the fovea. This will also result in sweeping of the peripheral scotomas across the scene at an angular speed related to that of the fixated object, which will be slower than that of more peripheral or farther static objects. The fixated object may be static as addressed above, but it may be moving within the environment such as another pedestrian walking in the same direction and speed as the patient, or in any other direction. If the fixated object is another pedestrian walking in the same direction and speed as the patient (see the second fixation marker in Video 4 (available at http://links.lww.com/OPX/A625) placed on the head of the person walking in the same direction as the patient), the pedestrian remains at the same retinal eccentricity. Fixating on the pedestrian means that the pedestrian image remains fixed on the patient’s retina while the whole environment is moving as the optic flow due to the patient’s walking. This situation is similar to the fixation at the focus of expansion except that movement of the environment across the scotoma may be faster on the farther side and slower on the other side if the pedestrian walking in the same direction is laterally off from the patient’s walking path. All these conditions are shown in Video 4 (available at http://links.lww.com/OPX/A625) illustrating the scotoma carving on a video of a patient walking in a virtual shopping mall with other pedestrians walking around in various directions and speeds. All the conditions described above are illustrated and if the observer is fixating on the marked fixation targets on the correct angular size display (100° horizontal span), the invisibility of the scotomas and the distortions caused by the carving in all these situations may be appreciated (Video 4, available at http://links.lww.com/OPX/A625). More complex interactions between the scotoma and other pedestrians occur when the patient is fixating a static target off the focus of expansion (gaze away from walking direction) which results in sweeping of the scotomas across the field of view while other pedestrians walking in various directions may interact with the “moving” scotomas.

**Carving a Central Scotoma**

Distortions due to the carving of peripheral scotomas were found to go largely unnoticed as the peripheral image content is out of the viewer’s attention, and the local spatial distortions are subjected to lower peripheral resolution and contrast sensitivities.^30^ We also evaluated the impact of scotoma carving applied centrally, representing the central field loss due to disciform macular degeneration where the full thickness of the retina is affected. Patients with central scotomas due to AMD also reported that they did not notice the scotomas as a black patch^1, 11^ though they frequently report distortions.^44-46^

In Fig. 6, we illustrate the effects of a central 6° diameter scotoma using the scotoma carving. The first observation is that there is no apparent black patch (Fig. 6B), which matches the patients’ reports. Without comparing Fig. 6B to the original image, Fig. 6A, it is difficult to notice the loss of the elided content at the center of the image. Due to the fixating (and thus the central scotoma) near the vanishing point of the scene, the converging roads after the scotoma carving appear natural without a careful examination or manual tracing of the lanes. The distortions around the carved-out scotoma are substantial, but both the distortions of the buildings or the road surfaces in Fig. 6B appear completely acceptable as architectural features seen in a geometric one-point (a central vanishing point) perspective. Fig. 6B shows the result of alternatingly carving with horizontal and vertical seams (see Appendix 1, available at http://links.lww.com/OPX/A621, for more details). Upon side-by-side comparison, the carved out missing contents (several buildings and trees) are easily noticeable.

All the processing and observations of the simulated central scotoma described above have been with high resolution (4096×3349) images examined with normal foveal vision. Some patients with macular scotoma describe distortions of architectural structures such as door frames or ceiling lines where the expected underlying structure is easy to compare to the appearance with the scotoma. They do, however, describe their vision, first and foremost, as “blurred” because they are forced to use their pericentral peripheral retina with its lower contrast sensitivity and lower resolution. When the eccentricity effect^32,42,43^ is applied to the original image (Fig. 6C) and then the high-resolution central vision is carved out, the carved image displays only the residual “blurred” pericentral vision of the reduced resolution outside the scotoma (Fig 6D). The lower resolution further masks, to some extent, spatial distortion, though not completely, as the degradation at this near periphery is not as severe as near the glaucoma arcuate scotomas.

A patient may be looking at the vanishing point of perspective as seen in Figs. 6A-D (i.e., the center of the image) or looking at different locations in the scene. Fig. 7E is illustrating the effect of such fixations away from the vanishing point on the appearance of the same scene with the same scotoma. When the fixation and the scotoma are shifted down and to the right (Fig. 7A), away from the vanishing point, the first effect of the scotoma carving (Fig. 7B) is the increased blur to the left and upper parts of the image due to the farther eccentricity from the fovea. The distortions surrounding the carved scotoma are more noticeable due to their difference of the local image details away from the vanishing point. Still, the distortions are not very apparent at that location. In Fig. 7C, the fixation is shifted up and to the right so that scotoma is carving a large portion of the high-rise building. The result of the scotoma carving is shown in Fig. 7D. In addition to the change in the blur which is increased here towards the lower left side of the image, the distortions here are very apparent as a deviation from the rectilinear shape of the building, both of the whole building and the grid-like structure of the windows. These effects illustrate the interaction of the local distortions with the specific image content. When the distorted areas are consisting of a so-called carpenter's world, the distortions are easier to notice and are more visible. This is consistent with reports of patients with central scotoma who describe distortions of architectural features such as ceiling lines and door frames (Appendix 2: Central Scotoma Carving, available at http://links.lww.com/OPX/A621).

**DISCUSSION**

Our interest here is focused on the invisibility of scotomas under natural daily viewing conditions. Numerous publications addressed the improved visibility of the scotoma under special (laboratory) conditions, as described below. The fact that special conditions were needed to enhance the visibility of the scotoma, or its boundary, using blinking^47^ or dynamic spatial noise^48^, indirectly demonstrates that the scotomas are in fact invisible under natural viewing conditions.

Sir David Brewster pointed out, as early as 1832, the surprising effect that a target falling in the optic nerve scotoma will disappear and be **replaced** with the color of the uniform background surrounding the target and the scotoma,^49^ This “filling-in” effect has been frequently described since then, and similar effects were documented for patients with discrete retinal lesions (i.e., Disciform macular scar,^50^ Chorioretinal scars from presumed toxoplasmosis^51^), or with cortical scotomas.^52^

Our scotoma carving hypothesis is distinct from, and conflicting with, the filling-in hypothesis that has been advanced to explain the phenomenon of the invisibility of the scotomas. The so-called filling-in phenomenon was reported to occur only in special scenarios where the scene under and around the scotoma is either a uniform, colored surface,^49, 53^ or a noise pattern.^54^ In the filling-in, the content under the scotoma is elided and then the field of view occupied by the scotoma (such a “hole”) is said to be replaced by the repeatable surrounding pattern. We note that filling-in with these scenarios is consistent with just carving out the elided content, without any replacement or filling-in. Thus, in the scotoma carving, nothing occupies the field of view of the scotoma (no “hole”) which is just simply removed from consciousness. The scene contents surrounding the scotoma are indistinguishable from the elided content in these special scenarios; a color surface remains a color surface, a noise pattern remains a noise pattern, and even periodic wallpaper-like patterns remain periodic patterns,^55, 56^ A carving out process without any replacement (filling-in) will similarly result in invisible scotomas for these types of spatial patterns.

The “filling-in” effect has been demonstrated with periodic patterns,^50, 57^ and static noise^50^ patterns. However, filling-in has not been reported to occur with complex natural scenes of everyday life, as we used here. We have found only one report that studied the filling-in effect with complex natural images or scenes on 10 patients with cortical scotomas^58^ and this report concluded “that filling-in is **impossible** for complex visual patterns”. In fact, no one has even suggested what filling-in effects in natural scene might look like.

The shrinking of lines passing through the invisible scotomas as demonstrated by Andrews^29^ and Safran^6^ is direct evidence for carving out and against filling-in. Under any type of filling-in, the length of the lines should not be changed. Whatever mechanisms that might cause filling-in when applied to a natural scene should create some false filling-in effects as it cannot fill in with the elided information based on the surrounding information (e.g., smaller detail than scotoma size). It has been demonstrated that the filling-in effect draws information from a very narrow ring stimuli surrounding the scotoma. Color filling-in was shown to be achieved by a mere 0.05° wide ring of color surrounding the physiological scotoma and by a texture ring of just 0.2° width at the edge of that scotoma.^59^ We are preparing a second manuscript on the invisibility of scotomas, which includes an actual filling-in effect, where complex image content from outside the scotoma is used to create spatially correlated content inside the scotoma (Scotoma Replacement, in preparation). This second process applies in cases of field loss due to photoreceptors loss. Our scotoma replacement process does account for the thin ring filling-in.^59^ But that process does not apply to scotomas due to loss at the retinal ganglion cell or higher neurons, which are addressed here.

A major difference between the scotoma carving hypothesis and the “filling-in” hypothesis is the different appearance of the image content that is not directly affected by the scotoma. The filling-in hypothesis does not require, nor does it allow any changes in the appearance of the content outside of the scotoma. In the filling-in, the “hole” in the image caused by the scotoma (i.e., the field of view covered by the scotoma) is filled-in and thus the space outside the scotoma should not be affected by that loss. It is similar to the content-aware filling algorithms such as the Magic Eraser applied in the Google Pixel 6 phone or the Object Eraser applied in the Samsung Galaxy phones, which result in distortion **only** in the applied area (i.e., scotoma). In the scotoma carving case, the removal of the elided content leaves a void in the image/scene, which when it is closed, as the carving process requires, pulls in the image content outside of the scotoma from both sides of the scotomas towards each other (the right side towards the left and vice versa, and the upper towards the lower, etc.) resulting in local spatial distortions. In applying the scotoma carving algorithm, the seams that are forced to pass through the scotoma diverge outside the scotoma resulting in the distortions reduced farther away from the scotoma (Fig 4B). That effect is apparent with the scotoma carving processing that removes whole seams. Because a typical discrete scotoma occupies a very small fraction of the visual field, the scotoma carving ends up with virtually no visible distortions farther away from the scotoma. All these phenomena are consistent with the description of distortions provided by patients.

Local distortions outside scotomas have been reported for central^1, 60, 61^ and peripheral scotomas.^62, 63^ The scotoma carving process resulted in local distortions outside the scotoma area, as we have shown here in a preliminary way. These may or may not be visible in peripheral locations or even centrally. The invisibility of local distortions in the normal periphery has been previously shown.^30, 31^ Results reported about the nature of peripheral scotoma distortions are not uniform across all studies and, therefore, not all are consistent with the effect of scotoma carving effects. Gerrits and Timmerman^51^ reported that for patients with AMD a slit of light going through the scotoma was never reported to be complete (a break in the line was noted). But Ramachandran^52, 64^ did find a completion of bright line across scotomas – though the process took a few seconds to occur. Zur et al.^50^ reported that two of their subjects observed a small break in the line going through their AMD scotomas while a third patient reported a complete line but with a central segment of lower contrast. When 6 such lines were combined into a grating pattern, they were seen as complete by all 3 subjects. Such line(s) through a scotoma will be completed by scotoma carving but may be interrupted depending on the angle of intersection with the scotoma border. A related effect was reported by Safran et al.^65^ where patients reported a missing section in the Amsler grid, which was, nevertheless, much smaller than the scotoma measured perimetrically. Yet, the same patients did not report such gaps when looking at natural images. Again, pointing to the nature of the Amsler grid as a perimetry device.

The missing (elided) content under a scotoma and at the borders of transitions between two residual areas around a scotoma are frequently barely noticeable with complex natural images after the application of the scotoma carving, even when the carved images are examined carefully. The loss of content is easier to note when the carving eliminates an object of interest (i.e., a face), but even in these situations, the visual system seems to be forgiving, especially when the effect is in peripheral vision or following saccadic eye movement as we demonstrated. Sergent,^66^ noted in the context of visual field loss that “perception is aimed at objects, not at patterns of light” leading to “intrinsic difficulty in attending to the pattern of light rather than to the object as such”.

The apparent invisibility or low visibility of scotoma borders that are brought into apposition by the carving process is similar to the low visibility of cuts in wallpaper.^56^ Wallpaper patterns are specifically designed to work well when carved out to fit the dimensions of the walls where the abutting cuts may not be matching sections of the pattern, yet they are not too disturbing to the eye, especially in peripheral vision. Similar designs are applied to cloth materials printed for shirt or dress manufacturing. The acceptability of such mismatched patterns to the visual system may be related to the amodal completion of occluded parts of objects. Amodal perception is the perception of the whole of an object when only parts of it affects the retina.^67^ The light pattern mismatches seen with the scotoma carving are similar to the light patterns at the edges of occluding surfaces. Occluding surfaces or parts of objects are very common in the environment, so they are familiar to the visual system and are not bothersome. For example, the carved-out parts of the cars in Fig. 4 appear to be occluded behind walls. The dynamic changes of occlusion and uncovering of such objects when one moves about in the environment are also very common and appear to be similar to the changes that occur at carved scotomas, as the simulated person is moving in the environment (see Video 4, available at http://links.lww.com/OPX/A625). Note that such optic flow movements are larger in magnitude in the peripheral vision where they are less noticeable because of the reduced resolution and contrast sensitivity at higher eccentricity.

Schuchard^57^ evaluated the perception of Amsler grid test patterns in patients with long standing bilateral macular scotomas. He found significant and substantial under-reporting of the scotoma, as also reported by Safran et. al.^65, 68^ The size of the area with no visible grid lines within an Amsler grid reported by patients was found to be much smaller than the scotoma mapped perimetrically. However, when not performing the Amsler grid task, these same patients did not perceive such gaps when looking at natural images. Our interest here is limited to the effects noted when observing natural scenes. The application of the scotoma carving algorithm is subject to numerous artefacts when applied to degenerate test stimuli such as the Amsler grid or a few lines.

The distortion reported by patients with disciform AMD and illustrated here has been interpreted by Massof to suggest a perceptual closure of visual space around the scotoma analogous to the closure of a draw string bag.^9^ This draw string closure hypothesis is similar but not identical to our scotoma carving. In particular, the cause and nature of the distortions outside the scotoma are different in nature in these two cases. The reports of these spatial distortions surrounding AMD central scotomas led to the design of field remapping vision aids using counter distortions.^9, 69-72^ These authors proposed that the distortion introduced around the scotomas may be fully or largely compensated by a remapping image processing. This aligns with the surround distortion after the scotoma carving and highlights the potential of (scotoma carving) simulations to aid in designing and evaluating vision rehabilitation devices and techniques. Yet, our finding of the low impact of the elided content and the practical invisibility of the distortions associated with the scotoma carving show that such treatments may not be very effective or valued, except perhaps for the central scotoma case. Even there the effect may be noticeable only in some cases, depending on local image content.

If an artificial scotoma (a square of uniform grey level) is placed over a repeated periodic grating background, the artificial scotoma is perceived to be replaced by the repeated pattern,^54^ but no such filling-in effect was found when the field of view surrounding the artificial scotoma was filled with square grid of random letters. An important distinction between the filling-in of natural scotoma (optic nerve head scotoma or pathological scotoma) and the artificial scotomas is that the filling-in of natural scotomas is apparent immediately upon testing, while filling-in phenomena with artificial scotomas take time to develop.^56,73-75^ The invisibility of monocular scotomas was reported immediately following the covering of the fellow healthy eye.^51, 76^ The scotoma invisibility phenomena discussed here also develops instantaneously. This aspect is particularly important when the interactions between the scotomas and eye movements are considered, as discussed below. Spatial distortion reported around central disciform scotomas caused, as a result of chorioretinal scaring following wet AMD, has been thought to be related to physical deformation of the retina near the scar at the scarring process.^77,78^ The scotoma carving process results in such distortions without postulating any retinal mechanical distortions (Fig. 6). The stipulation of retinal distortion as a cause for metamorphopsia assumes retinotopy mapping in the cortex. Indeed, such a mapping occurs at the lower levels of the cortex, but it is known to change toward object centered mapping at higher cortical centers.^79, 80^ More importantly, even at the lower cortical level, the retinotopy is neither simple nor linear. There is retinotopy in the gross sense that stimuli to the right of the fovea are mapped that way, but stimuli twice as far to the right are mapped farther to the right but may not necessarily be twice as far. Such relationships occur only with connected content. There are numerous examples of distorted spatial relationships under various conditions such as perisaccadic mis-localization,^81^ and compression^82^ and with numerous spatial illusions.

The simulation of the effect of retinal eccentricity on the resolution used here (Fig. 5C, Figs. 6 and 7) was applied using the pyramidal multiscale model applying measured contrast detection thresholds.^42^ That parameter-free model was used to generate simulations that were quantitatively verified for normal central vision of natural images.^83^ The peripheral vision eccentricity effect was added based on the measurement of contrast sensitivity across the retina and data from numerous other studies cited in Peli.^32^ The eccentricity model was fitted to derive a single eccentricity factor using experiments with complex images presented on a 64° wide display.^43^ Other computational models of vision across the visual field were proposed and applied to such simulations.^84-87^ However, verifications with complex images were not reported. When these simulations of peripheral vision were combined with representation of scotomas, the central scotomas were either left out of the presented images^32, 84^ or depicted as highly visible gray patches.^85^ Instead of eliminating the elided content under the scotoma, blurred versions of that content have been presented by Geisler and Perry^88^ and Jones et al.^89^ for glaucomatous arcuate scotomas, and by Goldstein et al.^35^ for central scotoma in AMD (using the Geisler’s algorithm). However, these processes use the image data from within the scotomas, which is not accessible to a visual system with the scotomas, and only blended/blurred that image content and did not fully delete the data from within the scotomas. In our scotoma carving process, the scotoma areas remain within the displayed image, and the image content covered by the scotoma is completely eliminated.

In considering visual simulations, *presenting images at the correct angular span is considered crucial if the observers are asked to observe the images for themselves*. The angular image size was explicitly addressed for the simulations presented by Capilla et al.^84^ and by Peli.^83, 90, 91^ The angular image size was explicitly manipulated by varying viewing distances in verifying the simulations.^83^ Wide peripheral images are difficult to display at the correct size. We used images cropped to the angular size of 45° or 60° so that readers can match the size on their display and thus experience the eccentricity effects in the (in)visibility of the scotoma. However, if the simulation is of peripheral scotomas that do not include the fovea, the relationship derived in Peli^90^ ensures that change in display angular size with distance maintains the effect of eccentricity on the content, enabling changes in viewing distance without disrupting the effect if readers maintain fixation. Only when the scotoma includes the fovea resulting in eccentric fixation being simulated, does the display size have to be maintained to veridically illustrate the effect of eccentricity.

**Clinical Significance**

Patients with glaucoma are not aware of their field loss, leading to calling the glaucoma the ‘silent thief'’ of sight.^92^ We have demonstrated and explained here that the patients do not gain awareness of the field loss even when saccadic eye movements result in uncovering objects from under the scotomas and covering other objects; nor with the gradual disappearing and reemerging of objects from the scotoma during smooth pursuit eye movements or due to optic flow when walking or driving. When the patients are told by their doctors that they have field loss, are shown the perimetric diagrams, as a proof, and see online the wrong common simulations with the black patches representing such loss, they immediately and easily determine that these illustrations do not represent their own visual perception.^10^ Understandingly, they conclude that all these issues do not apply to them. It is little wonder that these patients' compliance with drug treatments prescribed to them is very low.^93, 94^ It is likely that proper explanation of the invisibility of the field loss, as presented here, despite its complexity, will educate the patients about the nature of the disease and its visual effect and will encourage better compliance despite the “invisible” loss. Further, with such better explanations, patients may be taught to spot and monitor their “invisible” field loss and thus be more aware of the risk to their vision and the need for treatment.

Variety of training of eccentric fixation or modifying the preferred retinal locus were developed over the years. Many of these required awareness of the scotoma boundary and the location of the preferred retinal locus relative to the scotoma.^95^ Such performance would be impeded by the invisibility of the scotoma. In one study the outline of the scotoma was graphically presented one the functioning retina around the scotoma and patient were trained in reading with this artificial visibility of the scotoma. Only one of 8 patients with AMD that were used this as they practiced reading did not improved his reading speed following that brief practice.^96^ This is an example of the consideration of the invisibility of the scotoma directly in designing a rehabilitation technique/device.

**Limitations**

In this paper, we addressed only discrete relatively small scotomas surrounded by functional vision on all sides. The processing and analyses do not apply directly to other types of scotomas such as homonymous hemianopia, loss of the temporal crescent on one side^2^, and tunnel vision due to loss of peripheral field.^97^ In all these cases, the scotomas extend into the edge of the normal peripheral field. Understanding and simulating perception with these scotomas may require a different approach. However, these do share one clear characteristic with the discrete scotomas addressed here: the lack of conscious appreciation of the lost field. The fact that this occurs in these conditions is surprising and dramatic, especially when one considers that the central borders of the scotoma in many cases are very close to foveal vision and in some cases (HH) practically splitting the foveal field, yet they remain invisible. Another very dramatic demonstration of this effect is with RP patients with very narrow residual tunnel vision. We have seen in the lab 3 patients with just 5°, 10°, and 20° diameters of central residual fields, respectively, who reported driving regularly (this may be legal in some states). One of them who worked as a traveling salesman reported 4 minor collisions over the last year. Amazingly, all these patients denied any perceived restriction of their visual field. Currently, we do not know how to simulate these field losses in a way that is comparable to the scotoma carving.

The field of view outside the normal visual field (i.e., behind one’s head) is invisible. It clearly does not appear black or have any appearance at all, nor is there any transition noted between the seen field of view and the elided image content behind the head. That transition occurs at the far periphery where resolution and contrast sensitivity are both very poor, which may account for the invisibility of the transition. The transition from seeing to non-seeing visual field in hemianopia is reported to have similar perceptual characteristics.^66,98^ Despite taking place at field eccentricity with much higher resolution and contrast sensitivity, the missing field is not perceived as a black area (despite frequently being simulated this way), and the border or transition from seeing to non-seeing is not apparent. Similar effects are reported by patient with tunnel vision due to advanced retinitis pigmentosa or glaucoma. These conditions are outside of the scope of this paper and application of scotoma carving algorithm to these field losses does not yet contribute much to the understanding of the phenomenology.

**CONCLUSIONS**

The concept of scotoma carving presented here is indeed a hypothesis aimed at providing a plausible mechanistic illustration of the well-established facts that scotomas are not visible, not as black patches or in any other previously suggested ways. Our proposed scotoma carving was applied using the seam carving algorithm, but this is not intended to suggest this specific algorithm or model as the postulated physiological mechanism. The carving of elided content in the scotoma may not need any active process or mechanisms. The content may be simply ignored as is the scene behind our head.^53^ Rather, we suggest that the invisibility of spatio-temporal content within the scotoma results in a collapse of perceptual spatial relations across the scotoma. That process is, by definition, different from “filling-in”, and results in local distortions outside of the scotoma, unlike filling-in. Content-aware processing in the brain, similar in general nature to seam carving, would be an effective way of distributing and diffusing the distortions as the distance from the scotoma increases. Our preliminary presentation is strongly supported by the fact that numerous results in the literature regarding observations and studies with various discrete scotomas are consistent with the scotoma carving effects. The scotoma carving process itself has been developed only preliminarily and needs further work. Once that is completed or sufficiently advanced, we will be able to use the technique to create specific (and quantitative) predictions that may be tested with patients both in terms of appearance and perception as well as for quantitative performance on psychophysical tasks. We are currently piloting studies in which the existence of distortions of the type described qualitatively by patients with homonymous pericentral cortical scotomas^6^ will be documented and measured quantitatively.

**ACKNOWLEDGMENTS**

We thank Dr. Adam Reeves for numerous helpful discussions, comments, and suggestions regarding the writing of the article.

Supported in part by the National Institute of Health (NIH) grants R01-EY23385 (EP), EY30777 (JJ), and P30-EY003790 (SERI)

**APPENDICES AND SUPPLEMENTAL DIGITAL CONTENT**

Appendix 1: Scotoma Carving Computations, is available at http://links.lww.com/OPX/A621**.**

Appendix 2: The Invisibility of Jack-in-the-Box Effect with Peripheral Scotomas, is available at http://links.lww.com/OPX/A621**.**

Video 1 is available at http://links.lww.com/OPX/A622

Video 2 is available at http://links.lww.com/OPX/A623

Video 3 is available at http://links.lww.com/OPX/A624

Video 4 is available at http://links.lww.com/OPX/A625

**Supplementary material videos set 1:**

For each of the ten Arcuate Scotoma Videos, the following apply: Arcuate Scotoma videos 1-8 have their field of view set to 90°. The Arcuate Scotoma Wide 1 and Arcuate Scotoma Wide 2 videos have their field of view set to 110°. In each video, the field of view of the “flip book” displayed sections are 45°.The images following show the original image with scotomas outlines for each fixation point. The next set of images are the carved sections representing the view with saccades between centered fixations. The last static images enabled the viewer to carefully examine the carvings and distortions. The viewer may stop the video and examine the carvings and distortions at other fixations.

Supplemental Digital Content videos:

Arcuate Scotoma 1.mp4, is available at http://links.lww.com/OPX/A626

Arcuate Scotoma 2.mp4, is available at http://links.lww.com/OPX/A627

Arcuate Scotoma 3.mp4, is available at http://links.lww.com/OPX/A628

Arcuate Scotoma 4.mp4, is available at http://links.lww.com/OPX/A629

Arcuate Scotoma 5.mp4, is available at http://links.lww.com/OPX/A630

Arcuate Scotoma 6.mp4, is available at http://links.lww.com/OPX/A631

Arcuate Scotoma 7.mp4, is available at http://links.lww.com/OPX/A632

Arcuate Scotoma 8.mp4, is available at http://links.lww.com/OPX/A633

Arcuate Scotoma Wide 1.mp4, is available at http://links.lww.com/OPX/A634

Arcuate Scotoma Wide 2.mp4, is available at http://links.lww.com/OPX/A635

**Supplementary material videos set 2:**

For each of the following ten Central Scotoma Videos, the following apply: The horizontal field of view of all the original images is set to 60°. In each video, the first image shows the original image with scotoma outlines for all fixation points with preferred retinal locus marked on the lower right. The next set of images are the carved images representing the view with saccades between fixations. All images were processed to represent the eccentricity effect using the Pelimodel before carving. The authors added motion blurred frame between fixations to simulate the effect of saccadic blur.

Supplemental Digital Content videos:

Central Scotoma 1.mp4, is available at http://links.lww.com/OPX/A636

Central Scotoma 2.mp4, is available at http://links.lww.com/OPX/A637

Central Scotoma 3.mp4, is available at http://links.lww.com/OPX/A638

Central Scotoma 4.mp4, is available at http://links.lww.com/OPX/A639

Central Scotoma 5.mp4, is available at http://links.lww.com/OPX/A640

Central Scotoma 6.mp4, is available at http://links.lww.com/OPX/A641

Central Scotoma 7.mp4, is available at http://links.lww.com/OPX/A642

Central Scotoma 8.mp4, is available at http://links.lww.com/OPX/A643

Central Scotoma Wide 1.mp4, is available at http://links.lww.com/OPX/A644

Central Scotoma Wide 2.mp4, is available at http://links.lww.com/OPX/A645

**REFERENCES**

1. Schuchard RA. Adaptation to Macular Scotomas in Persons with Low Vision. Am J Occup Ther 1995;49:870-76.

2. Jung JH, Castle R, Kurukuti NM, et al. Field Expansion with Multiplexing Prism Glasses Improves Pedestrian Detection for Acquired Monocular Vision. Transl Vis Sci Technol 2020;9:1-15.

3. Peli E, Satgunam P. Bitemporal Hemianopia; Its Unique Binocular Complexities and a Novel Remedy. Ophthalmic Physiol Opt 2014;34:233-42.

4. Safran AB, Landis T. Plasticity in the Adult Visual Cortex: Implications for the Diagnosis of Visual Field Defects and Visual Rehabilitation. Curr Opin Ophthalmol 1996;7:53-64.

5. Safran AB, Duret F, Mermoud C, et al. Altered Perception of Distances with Homonymous Paracentral Scotomas. Vision Res 1996;36:375.

6. Safran AB, Achard O, Duret F, Landis T. The “Thin Man” Phenomenon: A Sign of Cortical Plasticity Following Inferior Homonymous Paracentral Scotomas. Br J Ophthalmol 1999;83:137-42.

7. Mavrakanas NA, Dang-Burgener NP, Lorincz EN, et al. Perceptual Distortion in Homonymous Paracentral Scotomas. J Neuroophthalmol 2009;29:37-42.

8. Dilks DD, Serences JT, Rosenau BJ, et al. Human Adult Cortical Reorganization and Consequent Visual Distortion. J Neurosci 2007;27:9585-94.

9. Massof RW. Smart Glasses [TV program]: PBS; 1993. Available at: <http://www.chedd-angier.com/frontiers/season4.html>. Accessed May 4, 2023.

10. Crabb DP, Smith ND, Glen FC, et al. How Does Glaucoma Look? Patient Perception of Visual Field Loss. Ophthalmology 2013;120:1120-26.

11. Taylor DJ, Edwards LA, Binns AM, Crabb DP. Seeing It Differently: Self-Reported Description of Vision Loss in Dry Age-Related Macular Degeneration. Ophthalmic Physiol Opt 2018;38:98-105.

12. Fletcher DC, Schuchard RA, Renninger LW. Patient Awareness of Binocular Central Scotoma in Age-Related Macular Degeneration. Optom Vis Sci 2012;89:1395-98.

13. Fujitani K, Su D, Ghassibi MP, et al. Assessment of Patient Perception of Glaucomatous Visual Field Loss and Its Association with Disease Severity Using Amsler Grid. PLoS One 2017;12:e0184230.

14. American Academy of Ophthalmology (AAO). Glaucoma Vision Simulator. Available at: <https://www.aao.org/eye-health/diseases/glaucoma-vision-simulator>. Accessed May 4, 2023.

15. Duffy M. Are We Using Accurate Images to Show What It Is Like to Have Macular Degeneration? In: VisionAware Blog: American Printing House for the Blind; 2017. Available at: <https://visionaware.org/blog/visionaware-blog/are-we-using-accurate-images-to-show-what-it-is-like-to-have-macular-degeneration-new-research-says-we-can-do-better/>. Accessed June 23, 2023.

16. Google. Google Search: Simulation of Visual Blind Spots. Available at: <https://www.google.com/search?q=simulation+of+visual+blind+spots&tbm=isch&ved=2ahUKEwj848WhrNz-AhVjFVkFHbvSD6oQ2-cCegQIABAA&oq=simulation+of+visual+blind+spots&gs_lcp=CgNpbWcQAzIECCMQJ1AAWABgqA5oAHAAeACAATmIATmSAQExmAEAqgELZ3dzLXdpei1pbWfAAQE&sclient=img&ei=eABUZPzHKeOq5NoPu6W_0Ao&bih=581&biw=1280&hl=en>. Accessed May 4, 2023.

17. Faye E. Peripheral Field Defects. In: Clinical Low Vision. Boston: Little Brown & Co., 1976:233-54.

18. Gottlieb DD. Living with Vision Loss. Atlanta, GA: St. Barthelemy Press, Ltd.; 1996.

19. Scheiman M, Scheiman M, Whittaker SG. Low Vision Rehabilitation: A Practical Guide for Occupational Therapists. Thorofare, NJ: Slack; 2007.

20. Welsh RC. The Roving Ring Scotoma: With Its Jack-in-the-Box Phenomenon of Strong-Plus (Aphakic) Spectacle Lenses. Am J Ophthalmol 1961;51:1277-81.

21. Boeder P. Spectacle Correction of Aphakia. Arch Ophthalmol 1962;68:870-74.

22. Jung JH, Kurukuti NM, Peli E. Photographic Depiction: The Perceived Scene through Spectacles-Mounted Low Vision Aids. Optom Vis Sci 2021;98:1210-26.

23. Apfelbaum HL, Ross NC, Bowers AR, Peli E. Considering Apical Scotomas, Confusion, and Diplopia When Prescribing Prisms for Homonymous Hemianopia. Transl Vis Sci Technol 2013;2, 1-18.

24. Peli E. Field Expansion for Homonymous Hemianopia by Optically-Induced Peripheral Exotropia. Optom Vis Sci 2000;77:453-64.

25. Jung JH, Peli E. Impact of High Power and Angle of Incidence on Prism Corrections for Visual Field Loss. Opt Eng 2014;53:1-13.

26. Satgunam P, Apfelbaum HL, Peli E. Volume Perimetry: Measurement in Depth of Visual Field Loss. Optom Vis Sci 2012;89:E1265-75.

27. Avidan S, Shamir A. Seam Carving for Content-Aware Image Resizing. ACM Trans Graph 2007;26:10.

28. Peli T, Malah D. A Study of Edge-Detection Algorithms. Comput Vision Graph 1982;20:1-21.

29. Andrews PR, Campbell FW. Images at the Blind Spot (Comment). Nature 1991;353:308.

30. Bex PJ. (In) Sensitivity to Spatial Distortion in Natural Scenes. J Vis 2010;10, 1-15.

31. Rovamo J, Makela P, Nasanen R, Whitaker D. Detection of Geometric Image Distortions at Various Eccentricities. Invest Ophthalmol Vis Sci 1997;38:1029-39.

32. Peli E, Yang J, Goldstein RB. Image Invariance with Changes in Size: The Role of Peripheral Contrast Thresholds. J Opt Soc Am (A) 1991;8:1762-74.

33. Thomas Pocklington Trust. How Do We See? How the world looks to blind and partially sighted people. Available at: <https://www.youtube.com/watch?v=BxqXEt5tuQs>. Accessed at May 4, 2023.

34.Legally Blind. See through My Eyes; a Low Vision Simulation; 2019. Available at: https://www.youtube.com/watch?v=xG7d-kIlnT8. Accessed at May 4, 2023.

35. Goldstein RB, Dugan E, Trachtenberg F, Peli E. The Impact of a Video Intervention on the Use of Low Vision Assistive Devices. Optom Vis Sci 2007;84:208-17.

36. Goldstein R, Peli E. Central Field Loss Simulation. 2022. Available at: https://pelilab.partners.org/lab/videos/cfl/CFL2_Simulation.wmv. Accessed at May 4, 2023.

37. Zeevi YY, Peli E. Smooth Eye-Movement Control with Secondary Visual Feedback. J Opt Soc Am (A) 1984;1:628-34.

38. Shanidze N, Fusco G, Potapchuk E, et al. Smooth Pursuit Eye Movements in Patients with Macular Degeneration. J Vis 2016;16:1-14.

39. Garcia-Pérez MA, Peli E. Visual Contrast Processing Is Largely Unaltered during Saccades. Front Psychol 2011;2:1-15.

40. Garcia-Pérez MA, Peli E. Intrasaccadic Perception. J Neurosci 2001;21:7313-22.

41. Luo G, Vargas-Martin F, Peli E. The Role of Peripheral Vision in Saccade Planning: Learning from People with Tunnel Vision. J Vis 2008;8:1-8.

42. Peli E. Contrast in Complex Images. J Opt Soc Am (A) 1990;7:2032-40.

43. Peli E, Geri GA. Discrimination of Wide-Field Images as a Test of a Peripheral-Vision Model. J Opt Soc Am (A) 2001;18:294-301.

44. Wiecek E, Lashkari K, Dakin SC, Bex P. Novel Quantitative Assessment of Metamorphopsia in Maculopathy. Invest Ophthalmol Vis Sci 2015;56:494-504.

45. Midena E, Vujosevic S. Metamorphopsia: An Overlooked Visual Symptom. Ophthalmic Res 2015;55:26-36.

46. Loewenstein A, Malach R, Goldstein M, et al. Replacing the Amsler Grid: A New Method for Monitoring Patients with Age-Related Macular Degeneration. Ophthalmology 2003;110:966-70.

47. Allen L, Folk JC, Thompson HS, Bourret JL. The Hole in My Vision: An Artist's View of His Own Macular Degeration. London: Penfield Press; 2000.

48. Aulhorn E, Kost G. [White Noise Field Campimetry. A New Form of Perimetric Examination]. Klin Monatsbl Augenheilkd 1988;192:284-88.

49. Brewster D. Letters on Natural Magic; Addressed to Sir Walter Scott, Bart. London: John Murray; 1832.

50. Zur D, Ullman S. Filling-in of Retinal Scotomas. Vision Res 2003;43:971-82.

51. Gerrits HJ, Timmerman GJ. The Filling-in Process in Patients with Retinal Scotomata. Vision Res 1969;9:439-42.

52. Ramachandran VS. Perceptual "Filling in" of the Blind Spot and of Cortical and Retinal Scotomas. Invest Ophthalmol Vis Sci 1992;33:1348.

53. Anstis S. Visual Filling-In. Curr Biol 2010;20, 1-3.

54. Ramachandran VS, Gregory RL. Perceptual Filling in of Artificially Induced Scotomas in Human Vision. Nature 1991;350:699-702.

55. Ramachandran VS. Filling in Gaps in Perception: Part Ii. Scotomas and Phantom Limbs. Curr Dir Psychol Sci 1993;2:56-65.

56. Ramachandran VS. Forward. In: Pessoa L, De Weerd P, eds. Filling-In: From Perceptual Completion to Cortical Reorganization. New York: Oxford University Press, 2003:xi-xxii.

57. Schuchard RA. Validity and Interpretation of Amsler Grid Reports. Arch Ophthalmol 1993;111:776-80.

58. Bachmann G, Fahle M. Filling-in Mechanisms for Different Components of Perception in Patients with Visual-Field Defects. Perception 1995;24:49.

59. Spillmann L, Otte T, Hamburger K, Magnussen S. Perceptual Filling-in from the Edge of the Blind Spot. Vision Res 2006;46:4252-57.

60. Wittich W, Overbury O, Kapusta MA, et al. Macular Hole: Perceptual Filling-in across Central Scotomas. Vision Res 2006;46:4064-70.

61. Wittich W, Overbury O, Kapusta MA, Faubert J. Visual Function Assessment and Metamorphopsia after Macular Hole Surgery. Ophthalmic Physiol Opt 2005;25:534-42.

62. Wittich W, Watanabe DH, Kapusta MA, Overbury O. Subjective Perception of Visual Distortions or Scotomas in Individuals with Retinitis Pigmentosa. J Vis Impair Blind 2011;105:50-5.

63. Wittich W, Faubert J, Watanabe DH, et al. Spatial Judgments in Patients with Retinitis Pigmentosa. Vision Res 2011;51:165-73.

64. Ramachandran VS. Filling in Gaps in Perception: Part I. Curr Dir Psychol Sci 1992;1:199-205.

65. Safran AB, Landis T. A Simple Technique for Routine Evaluation of the Filling-in Phenomenon in Clinical Practice. Neuroophthalmology 1996;16.

66. Sergent J. An Investigation into Perceptual Completion in Blind Areas of the Visual Field. Brain 1988;111:347-73.

67. Greene HH, Brown JM. Amodal Completion and Localization. Vision Res 2000;40:383-90.

68. Achard OA, Safran AB, Duret FC, Ragama E. Role of the Completion Phenomenon in the Evaluation of Amsler Grid Results. Am J Ophthalmol 1995;120:322-29.

69. Loshin DS, Juday RD. The Programmable Remapper: Clinical Applications for Patients with Field Defects. Optom Vis Sci 1989;66:389-95.

70. Wensveen JM, Bedell HE, Loshin DS. Reading Rates with Artificial Central Scotomata with and without Spatial Remapping of Print. Optom Vis Sci 1995;72:100-14.

71. Gupta A, Mesik J, Engel SA, et al. Beneficial Effects of Spatial Remapping for Reading with Simulated Central Field Loss. Invest Ophthalmol Vis Sci 2018;59:1105-12.

72. Deemer AD, Bradley CK, Ross NC, et al. Low Vision Enhancement with Head-Mounted Video Display Systems: Are We There Yet? Optom Vis Sci 2018;95:694-703.

73. Caputo G. Texture Brightness Filling-In. Vision Res 1998;38:841-51.

74. Arrington KF. The Temporal Dynamics of Brightness Filling-In. Vision Res 1994;34:3371-87.

75. Spillmann L, De Weerd P. Mechanisms of surface completion: perceptual filling-in of texture. In: Pessoa L, De Weerd P, eds. Filling-In: From Perceptual Completion to Cortical Reorganization. New York: Oxford University Press, 2003:81-106.

76. Jamal YA, Dilks DD. Rapid Topographic Reorganization in Adult Human Primary Visual Cortex (V1) during Noninvasive and Reversible Deprivation. Proc Nat Acad Sci U S A 2020;117:11059-67.

77. Kashani AH, Aaberg TM, Jr., Capone A, Jr. Vitreomacular Traction as a Consequence of Posterior Hyaloidal Contraction after Transpupillary Thermotherapy. Am J Ophthalmol 2013;155:937-45.

78. Tong B, Wang C, Qi X. Unusual Rapid Resolution of Postsclerectomy Exudative Retinal Detachment with Topical NSAIDs Therapy in a Case of Nanophthalmos. J Int Med Res 2019;48.

79. Larsson J, Heeger DJ. Two Retinotopic Visual Areas in Human Lateral Occipital Cortex. J Neurosci 2006;26:13128-42.

80. Huk AC, Dougherty RF, Heeger DJ. Retinotopy and Functional Subdivision of Human Areas MT and MST. J Neurosci 2002;22:7195-205.

81. Luo G, Garaas T, Pomplun M. Salient Stimulus Attracts Focus of Peri-Saccadic Mislocalization. Vision Res 2014;100:93-8.

82. Luo G, Garass T, Pomplun M, Peli E. Peri-Saccadic Mislocalization Centered at Salient Stimulus Instead of Saccade Goal. J Vis 2010;10:519.

83. Peli E. Contrast Sensitivity Function and Image Discrimination. J Opt Soc Am (A) 2001;18:283-93.

84. Capilla P, Luque MJ, de Fez MD, et al. Simulating Images Seen by Patients with Inhomogeneous Sensitivity Losses. Optom Vis Sci 2012;89:1543-56.

85. Marmor DJ, Marmor MF. Simulating Vision with and without Macular Disease. Arch Ophthalmol 2010;128:117-25.

86. Geisler WS, Perry JS. Real-Time Foveated Multiresolution System for Low-Bandwidth Video Communication. Photonics West '98 Electronic Imaging 1998;3299:294-305.

87. Haun AM. What Is Visible across the Visual Field? Neurosci Conscious 2021;2021:niab006.

88. Geisler WS, Perry JS. Real-Time Simulation of Arbitrary Visual Fields. ETRA '02: 2002 Symposium on Eye Tracking Research & Applications 2002:83-7.

89. Jones PR, Somoskeoy T, Chow-Wing-Bom H, et al. Seeing Other Perspectives: Evaluating the Use of Virtual and Augmented Reality to Simulate Visual Impairments (OpenVisSim). NPJ Digit Med 2020;3:32.

90. Peli E, Goldstein RB, Young GM, et al. Image Enhancement for the Visually Impaired: Simulations and Experimental Results. Invest Ophthalmol Vis Sci 1991;32:2337-50.

91. Peli E. Limitations of Image Enhancement for the Visually Impaired. Optom Vis Sci 1992;69:15-24.

92. National Eye Institute (NEI). Glaucoma: The 'Silent Thief' Begins to Tell Its Secrets. In: NRI Research News; 2014. Available at: <https://www.nei.nih.gov/about/news-and-events/news/glaucoma-silent-thief-begins-tell-its-secrets>. Accessed June 23, 2023.

93. Granstrom PA. Progression of Visual Field Defects in Glaucoma. Relation to Compliance with Pilocarpine Therapy. Arch Ophthalmol 1985;103:529-31.

94. Kass MA. Compliance and Prognosis in Glaucoma. Arch Ophthalmol 1985;103:504.

95. Holcomb JG, Goodrich GL. Eccentric Viewing Training. J Am Optom Assoc 1976;47:1438-43.

96. Pratt JD, Stevenson SB, Bedell HE. Scotoma Visibility and Reading Rate with Bilateral Central Scotomas. Optom Vis Sci 2017;94:279-89.

97. Apfelbaum H, Peli E. Tunnel Vision Prismatic Field Expansion: Challenges and Requirements. Transl Vis Sci Technol 2015;4:1-13.

98. Gassel MM, Williams DR. Visual Function in Patients with Homonymous Hemianopia. Iii. The Completion Phenomenon; Insight and Attitude to the Defect; and Visual Functional Efficiency. Brain 1963;86:229-60.

**FIGURE LEGENDS**

**Figure 1.** The “invisibility” of perimetrically recorded optical scotomas. **(A)** Monocular (right eye) perimetry of a normally sighted person wearing a +10.00D lenticular spectacle lens (39 mm diameter lenticular). **(B)** A photo taken by a camera held behind the same lens depicting the user's view.^22^ The magnification ring scotoma recorded in (A) is only notable in (B) by noticing the missing content (missing parts of the entrance on the right and missing parts of posters) between the (magnified) view through the lens and the view outside the lens. **(C)** Monocular (left eye) perimetry from a patient with left homonymous hemianopia wearing upper and lower horizontal peripheral prisms (40Δ≈20°) on the left lens. The apical scotomas are easily measurable on the right seeing side, in addition to the expanded field of view measured to the left. **(D)** A photograph of the view through the peripheral prisms lens.^25^ For a patient with left hemianopia, the left half of this image will not be visible, except as seen through the right side of the prisms. The apical scotomas in (C) are not explicitly noticeable but can be appreciated by noting that the portions of the scene (i.e., the person’s face) are not visible, in or out of the prism, as it is replaced by the view through the prism of the portions of the scene farther to the left.

**Figure 2.** The invisibility of the scotoma from bitemporal hemianopia combined with left esotropia. **(A)** A schematic drawing of a chiasmal lesion causing a vertical scotoma (marked as black), as wide as the esotropia angle. The inset shows a dichoptic field diagram with the loss of both peripheral crescents and a vertical central scotoma. **(B)** A typical (**incorrect**) simulation of the field loss is illustrated as a black stripe in the middle of the scene that matches the field diagram in (A). **(C)** The actual view of the scene as perceived by such a patient with bitemporal hemianopia.^3^ Due to the elided contents at the center (under the black strip in Fig. 2B), small mismatches may be detected across the vertical meridian under careful examination. **(D)** Photographic depiction of a similar effect of optical scotoma created by slightly misaligned wall mirrors found in a bathroom. Although no scotoma or black vertical strip is explicitly visible (except for the thin line between the mirror boundaries), the missing elided image content (nose) due to the scotoma can be inferred cognitively.

**Figure 3.** The ‘thin man’^6^ perceived by patients with cortical paracentral field loss. **(A)** Field diagram of a small (5° x 8°) bilateral homonymous paracentral scotoma. **(B)** The binocular scotoma mask is shown over the father’s left shoulder. The 6 first seams to be removed in scotoma carving are annotated in colors. These seams are converging into the scotoma from above and then diverging below the scotoma. **(C)** The scotoma carving shrank the left shoulder. The scotoma itself is invisible. Additional local distortions (i.e, pots on the stove, lines on the shirt, and the narrower wall of the furnace) are only noticeable by carefully comparing (B) and (C).

**Figure 4.** Scotoma carving simulation of vision with paracentral arcuate scotomas from glaucoma (eccentricity of ~15°). **(A)** Typical vision with scotoma simulation (3 black paracentral patches with the black cross with white border marking the associated fixation position) adapted from Crabb,^10^ image reproduced with permission, Superimposed yellow and red outlines indicate the location of those scotomas at two different fixation locations (yellow and red crosses), representing saccadic eye movements. Scotoma carving simulation results in the removal of objects under the scotomas marked in (A) by the dashed yellow outlines **(B)** and the solid red lines **(C)**. The local spatial distortions are not easily noted when maintaining fixation at the cross. The distortions pop out as motion artefacts when images in (B) and (C) are abruptly flipped (see Video 1, available at http://links.lww.com/OPX/A622). With guided-fixations and blurred frames inserted between fixations as simulation of saccadic blur, the motion, and the distortions and thus scotomas are invisible (see Video 2, available at http://links.lww.com/OPX/A623).

**Figure 5.** “Flipbook” simulations of saccadic effect presentation with scotoma carving. **(A)** a panorama image of the full farmhouse scene spanning 109°, which is too large to be presented on most displays. The 10 small crosses (yellow, green, and blue) mark the selected locations of fixation implemented in the video frames. For two of the 10 fixations, we show the positions of the corresponding arcuate scotomas carved out (blue and green). **(B)** A sample frame from the green fixation with corresponding green arcuate scotomas shown in (A) carved out. When fixating on the center fixation mark, it is hard to see even the large distortions on the right and almost impossible to note the distortion caused by the carving on the left. **(C)** Another frame of the flipbook corresponding to the blue arcuate scotoma in (A) was first processed to simulate the loss of resolution as a function of eccentricity^32^ and then carved out. In both (B) and (C) and all fixations in Video 3 (available at http://links.lww.com/OPX/A624), it is almost impossible to see the effects of the smaller lower scotoma. The observer may stop the video at any frame and examine the whole frame foveally. This should enable the detection of the effects of scotoma carving including some dramatic distortions that are not visible when fixating centrally.

**Figure 6.** Central scotoma carving. **(A)** A scene spanning 32° with the location of a 6° central disciform scotoma marked (yellow circle). **(B)** Alternating scotoma carving. Applying vertical seam alternating with horizontal seam, to the original scene in (A). Distortions are apparent in comparing the images in (A) and (B), but the image in (B) is completely acceptable if examined without comparison. (C) The original image in (A) was processed with the Peli model to simulate the decline in resolution and contrast sensitivity with retinal eccentricity^32, 43^. (D) The image in (C) following alternating scotoma carving representing the view of a patient with a disciform scotoma. The most notable effect is the “blurriness” of the view, as reported by patients. The central elided content from (C) is invisible here. (E) The image in (C) with the fixation and scotoma position shifted 6.5° down and 8° rightward from the center. Note the increased blur near the left edge of the image and decreased blur on the lower right in comparison with (C).

Figure 7. (A) The image in (Fig. 6C) with the fixation and scotoma position shifted 6.5° down and 8° rightward from the center. Note the increased blur near the left edge of the image and decreased blur on the lower right in comparison with (Fig. 6C). (B) The image in (A) following alternating scotoma carving. The distortions become more apparent even without comparison with the image in (A) because they are not at or near the perspective vanishing point. (C) The image in (Fig. 6C) with the fixation and scotoma position shifted 6.5° up and 10° rightward from the center to fall onto the high-rise building. (D) The image in (C) following alternating scotoma carving. Note the increased blur on the left and below in this image in comparison with (Fig. 6C). The distortions here are apparent even without comparison with the image in (A) because they affect the windows grid pattern in addition to not being near the perspective vanishing point. The angular size of the printed images is much smaller than the simulated size. The reader may enlarge each image to fill the page by zooming in. Even at that scale, the images are only about 65% of the actual angular size.
